# Discovery and Structure-Based Optimization of 6-Bromotryptamine Derivatives as Potential 5-HT_2A_ Receptor Antagonists

**DOI:** 10.3390/molecules200917675

**Published:** 2015-09-23

**Authors:** Lijian Ding, Shan He, Wei Wu, Haixiao Jin, Peng Zhu, Jinrong Zhang, Tingting Wang, Ye Yuan, Xiaojun Yan

**Affiliations:** 1 Laboratory of Marine Natural Products, School of Marine Sciences, Ningbo University, Ningbo 315211, China; E-Mails: huahua20062008@126.com (L.D.); jinhaixiao@nbu.edu.cn (H.J.); zhangjinrong@nbu.edu.cn (J.Z.); happyjelly@yeah.net (T.W.); 23yuanye@163.com (Y.Y.); 2 College of Pharmacy, Jinan University, Guangzhou 510632, China; 3 Collaborative Innovation Center for Zhejiang Marine High-efficiency and Healthy Aquaculture, Ningbo 315211, China; 4 Key Laboratory of Applied Marine Biotechnology of Ministry of Education, Ningbo University, Ningbo 315211, China; E-Mails: wuweixiehou@163.com (W.W.); zhupeng@nbu.edu.cn (P.Z.)

**Keywords:** 6-bromotryptamine, *Pseudoalteromonas rubra*, 5-HT_2A_ receptor, antagonist

## Abstract

5-Hydroxytryptamine type 2A (5-HT_2A_) receptor is an important target for developing innovative antipsychotic agents in neuropsychiatric disorder therapies. To search for 5-HT_2A_ receptor antagonists, a new indole alkaloid termed 6-bromo-*N*-propionyltryptamine (**1**), together with one known homologue 6-bromo-*N*-acetyltryptamine (**2**) were isolated and identified from a marine bacterium *Pseudoalteromonas rubra* QD1-2. Compound **1** with an *N*-propionyl side chain exhibited stronger 5-HT_2A_ receptor antagonist activity than that of *N*-acetyl derivative (**2**), indicating that 6-bromotryptamine analogues with a longer chain acyl group perhaps displayed a more potent capacity to the target. Therefore, a series of new 6-bromotryptamine analogues (**3**–**7**) with different chain length of the acyl group (C4–C8) were prepared and evaluated activity against 5-HT_2A_ receptor. Remarkably, 6-bromo-*N*-hexanoyltryptamine (**5**) displayed the most effective inhibitory activity, which was 5-fold stronger than that of the parent compound **1** and showed 70% efficacy of the positive control (ketanserin tartrate).

## 1. Introduction

Serotonin or 5-hydroxytryptamine (5-HT) is a monoamine neurotransmitter that modulates a wide range of physiological and behavioral functions of the central nervous system (CNS), including cognition, mood, mating, feeding, and sleeping [1,2,3]. 5-HT receptors are members of the G-protein-coupled receptor superfamily, which are categorized into seven major families, 5-HT_1−7_. Among them, the 5-HT_2_ receptor subfamily contains three members, namely 5-HT_2A_, 5-HT_2B_, and 5-HT_2C_. Extensive studies have revealed that the 5-HT_2A_ receptor played a critical role in the regulation of neuropsychiatric disorders associated with depression, Parkinson’s and Alzheimer’s disease [4,5,6,7]. Hence, a 5-HT_2A_ receptor antagonist is therapeutically relevant for neurological disorders. For instance, M100907 with the 5-HT_2A_ antagonist activity has entered onto the phase II clinical trial for the treatment of schizophrenia [8].

**Figure 1:**
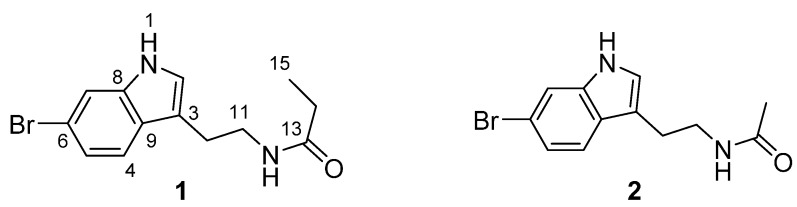
Structures of **1** and **2** from *P. rubra* QD1-2.

**Scheme 1:**
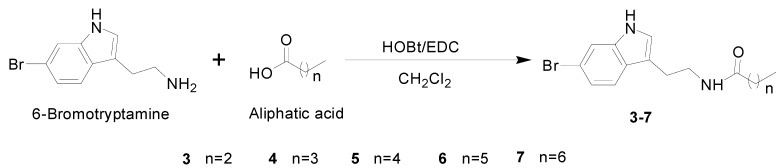
Synthesis of 6-bromotryptamine derivatives **3**–**7**.

In our continuing program on the discovery of 5-HT_2A_ receptor antagonists from marine bacterial culture broths, the EtOAc extract from *Pseudoalteromonas rubra* QD1-2 displayed promising activity. Bioassay-guided isolation procedures led to the obtaining of 6-bromo-*N*-propionyltryptamine (**1**) and 6-bromo-*N*-acetyltryptamine (**2**) (Figure 1). Intriguingly, the new compound (**1**) with *N*-propionyl side chain showed a higher inhibitory activity than that of *N*-acetyl derivative (**2**), thus revealing that a longer chain acyl group attached to 6-bromotryptamine probably possessed a better inhibition capacity to 5-HT_2A_ receptor. In order to test this hypothesis, a new series of 6-bromotryptamine derivatives with a longer chain length than that of compounds **1** and **2**, were synthesized (**3**–**7**, Scheme 1) and evaluated the preliminary structure-activity relationships (SAR). Consequently, compound **5** with *N*-hexanoyl side chain was identified as the most potent one in the series of 6-bromotryptamine scaffold. Herein, we report the isolation, identification, synthesis, and SAR of 6-bromotryptamine derivatives.

## 2. Results and Discussion

### 2.1. Structure Elucidation of 6-Bromotryptamine Derivatives **1**–**2**

**Figure 2:**
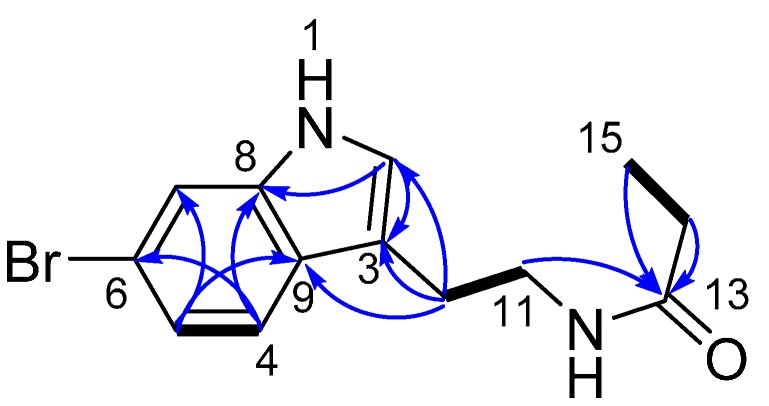
Key ^1^H-^1^H COSY (bold bonds) and HMBC (arrows) correlations of **1**.

Compound **1** was obtained as a yellowish powder. The molecular formula was established as C_13_H_15_BrN_2_O by high-resolution electrospray ionization mass spectroscopy (HRESIMS), which gave equal-intensity ions at *m*/*z* 317.0269/319.0254 [M + Na]^+^, suggesting **1** contained one bromine atom. The ^13^C-NMR spectrum of **1** showed signals for one amide carbonyl group at δ_C_ 177.0 (C-13), four sp^2^ quaternary carbons at δ_C_ 113.8 (C-3), 115.7 (C-6), 127.3 (C-9), 138.9 (C-8), four sp^2^ methines at δ_C_ 115.1 (C-7), 120.8 (C-4), 122.7 (C-5), 124.3 (C-2), three methylenes at δ_C_ 26.1 (C-10), 43.3 (C-11), and one methyl at δ_C_ 10.5 (C-15). The ^1^H-NMR signals at δ_H_ 7.07 (1H, s, H-2), 7.10 (1H, dd, *J* = 8.5, 1.8 Hz, H-5), 7.47 (1H, d, *J* = 8.4 Hz, H-4), and 7.48 (1H, d, *J* = 1.8 Hz, H-7) displayed a classic pattern for a 3,5- or 3,6-disubstituted indole moiety, which was supported by the HMBC correlations of H-2/C-3 and C-8, H-4/C-6 and C-8, H-5/C-7 and C-9, and H-7/C-5 and C-9 (Table 1). Furthermore, these HMBC connectivities together with the COSY correlation of H-4/H-5, showed the location of the bromine atom at C-6, which was finally determined by comparison of the observed chemical shifts with those reported for 5 or 6-bromoindole-containing compounds [9,10]. In addition, the COSY correlation of H-10 (δ_H_ 2.91, t, *J* = 7.2 Hz)/H-11 (δ_H_ 3.44, t, *J* = 7.3 Hz) for **1** disclosed the presence of a pair of coupled methylene groups, which was attached to the 6-bromoindole moiety at C-3 due to the HMBC correlations of H-10/C-2 and C-3 and H-11/C-3, thus forming a 6-bromotryptamine moiety (Figure 2). The remaining spectroscopic features of **1** indicated the presence of a propionyl group based on the COSY correlation of H-14 (δ_H_ 2.16, q, *J* = 7.6 Hz)/H-15 (δ_H_ 1.09, t, *J* = 7.7 Hz) and HMBC correlations of H-14 and H-15/C-13 (Figure 2). Finally, the propionyl group was connected to 6-bromotryptamine moiety through an amide bond by the HMBC correlation of H-11/C-13. According to the aforementioned information, the structure of **1** was unambiguously assigned as 6-bromo-*N*-propionyltryptamine.

Compound **2** was a known synthetic compound but now isolated for the first time as a natural product. Its structure was determined as 6-bromo-*N*-acetyltryptamine, which was confirmed by comparing their spectral data with the reference reported [11].

**Table 1:** ^1^H- and ^13^C-NMR, COSY, and HMBC Data for **1** in CD_3_OD.

Position	δ_C_, Type	δ_H_ (*J* in Hz)	COSY	HMBC (H→C)
2	124.3, CH	7.07, s		3, 8
3	113.8, C			
4	120.8, CH	7.47, d (8.4)	5	6, 8
5	122.7, CH	7.10, dd (8.5, 1.8)	4	7, 9
6	115.7, C			
7	115.1, CH	7.48, d (1.8)		5, 9
8	138.9, C			
9	127.3, C			
10	26.1, CH_2_	2.91, t (7.2)	11	2, 3, 9, 11
11	41.3, CH_2_	3.44, t (7.3)	10	3, 10,13
13	177.0, C			
14	30.3, CH_2_	2.16, q (7.6)	15	13, 15
15	10.5, CH_3_	1.09, t (7.7)	14	13, 14

### 2.2. Syntheses of 6-Bromotryptamine Derivatives **3**–**7**

The synthetic route of **3**–**7** is depicted in Scheme 1, which was carried out by the condensation of 6-bromotryptamine and butyric acid, pentanoic acid, hexanoic acid, heptanoic acid and octanoic acid, respectively, using 1-(3-dimethylaminopropyl)-3-ethylcarbodiimide hydrochloride (EDC·HCl) and hydroxybenzotriazole (HOBt) in dichloromethane (DCM) solution at room temperature for 12 h [12,13,14]. Compounds **3**–**7** were synthesized for the first time in yields ranging from 79% to 85%, the structures of which were identified and characterized by ESI-MS and NMR spectroscopy .This method was proved to provide a rapid, and high yielding procedure to afford acyl chain derivatives of 6-bromotryptamine.

### 2.3. Structure-Activity Relationships

**Figure 3:**
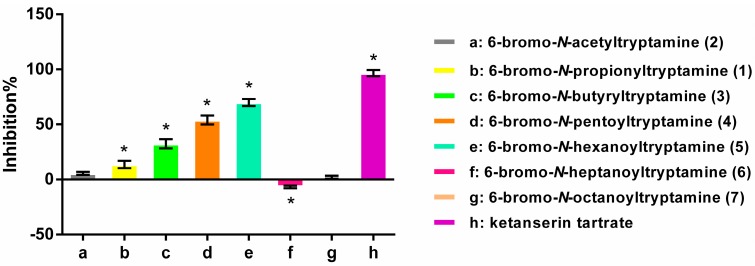
5-HT_2A_ receptor antagonist activity of 6-bromotryptamine derivatives (10 μM). All values are expressed as means ± SD (*n* = 3) and * indicates significant at *p* < 0.05 relative to compound **2**. Ketanserin tartrate was used as positive control.

The 6-bromotryptamine derivatives (**1**–**7**) were evaluated at a concentration of 10 μM for their antagonist activity on 5-HT_2A_ receptor detectable by calcium flux assay. The results (Figure 3) highlighted the importance of the length of the acyl chain in the 6-bromotryptamine derivatives for the antagonist activity. Interestingly, we observed that the activity gradually improved in response to increasing chain length (C2–C6), but a reverse tendency occurred when the chain length was C7 or C8. 6-Bromo-*N*-hexanoyltryptamine (**5**) thus exhibited an approximately 70% inhibition activity compared with the positive control, which was the most potency in the series. Notably, compound **5** displayed a roughly 4-fold higher inhibitory activity than the parent analogue **1**. These observations might be explained by two mechanisms. A longer acyl side chain related to a higher lipophilicity, has the positive consequence in membrane permeation, probably eliciting more effective inhibitory potency for the target. However, the bulk chain (when > C6) caused an acyl group-dependent decrease in the binding affinity, and likely reduced the inhibitory potency for 5-HT_2A_ receptor [15].

## 3. Experimental Section

### 3.1. General Experimental Procedures

Preparative HPLC equipment was carried out on a Waters 600 pump equipped with a Waters model 2996 diode array detector and Waters Empower System (Waters Co., Milford, CT, USA). HRESIMS analysis was recorded using a triple quadrupole coupled with time-of-flight mass spectrometry with an ESI interface (Waters Co.). ESI-MS analysis was analyzed using a triple quadrupoles mass spectrometer (Thermo, San Jose, CA, USA). NMR experiments were performed using Bruker AVANCE 400 and 500 MHz NMR spectrometers (Bruker, Karlsruhe, Germany). A Sun-Fire C18 column (20 × 250 mm, ID. 5 μm) was used for reverse-phase separations. All solvents were HPLC grade (Shanghai ANPEL Scientific Instrument, Shanghai, China). 6-Bromotryptamine was purchased from Lianchuang Company (Anhui Lianchuang Pharmceutical Chemistry Co., Ltd., Hefei, China). All aliphatic acids, *N*-(3-(dimethylamino) propyl)-*N*-ethylcarbodiimide hydrochloride (EDC·HCl), and hydroxybenzotriazole hydrate (HOBt) were purchased from Aldrich Chemicals Company (Milwaukee, WI, USA). Calcium flux assay was performed on Fluorescence Image Plate Reader (Molecular Devices, San Francisco, CA, USA) with a FLIPR calcium assay kit. Ketanserin tartrate was purchased from Tocris Cookson Inc. (Ellisville, MO, USA). CHO-K1 (Chinese hamster ovary) was purchased from GenScript Corporation (Piscataway, NJ, USA).

### 3.2. Bacterial Material and Fermentation

The strain *P. rubra* QD1-2 was isolated from a seawater sample collected from Qingdao Sea area in China, which was stored in China General Microbiological Culture Collection Center (CGMCC, No. 6555). The fermentation was implemented in two steps: seed growth and scale-up fermentation. For the seed growth stage, *P. rubra* QD1-2 from a plate culture was inoculated into 100 mL of the basic seed medium (peptone 1 g/L, yeast extract 2.5 g/L, glucose 0.1 g/L dissolved in seawater, pH 7.0) in a 500 mL Erlenmeyer flask and grown in a shaker incubator at 25 °C and 150 rpm for 24 h. Then, seed cultures were inoculated into 1 L Erlenmeyer flasks containing 400 mL of fermentation medium (peptone 1 g/L, yeast extract 2.5 g/L, glucose 0.1 g/L dissolved in seawater, pH 7.0). The strain was fermented on rotary shakers at 25 °C with 150 rpm for 7 days.

### 3.3. Extraction and Isolation

A total of the 20 L fermentation broth was harvested and filtered by centrifuge and extracted with EtOAc three times. The extract was concentrated under reduced pressure on a rotary evaporator at 37 °C, which was combined to obtain 1.1 g of crude extract. The extract was directly purified by preparative-HPLC with an isocratic elution of 65% MeOH/H_2_O (Flow 8 mL/min, UV detection at 280 nm) to afford compounds **1** (12 mg) and **2** (14 mg).

*6-Bromo-N-propionyltryptamine* (**1**): Yellowish powder; The molecular formula of **1** (HRESIMS *m*/*z* 317.0269, 319.0254 [M + Na]^+^, calc. for C_13_H_15_BrN_2_O); ^1^H- and ^13^C-NMR data in CD_3_OD, see Table 1.

*6-Bromo-N-acetyltryptamine* (**2**): Yellowish powder; ^1^H-NMR (400 MHz, CD_3_OD): δ (ppm) 7.49 (d, *J* = 1.5 Hz, 1H, 7-H), 7.45 (d, *J* = 8.4 Hz, 1H, 4-H), 7.10 (dd, *J* = 8.4, 1.6 Hz, 1H, 5-H), 7.1 (s, 1H, 2-H), 3.44 (t, *J* = 7.4 Hz, 2H, 11-H), 2.90 (t, *J* = 7.1 Hz, 2H, 10-H), 1.90 (s, 3H, 14-H); ^13^C-NMR (100 MHz, CD_3_OD) δ (ppm) 173.22 (qC, C-13), 138.91 (qC, C-8), 127.78 (qC, C-9), 124.31 (CH, C-2), 122.69 (CH, C-5), 120.71 (CH, C-4), 115.73 (qC, C-6), 115.07 (CH, C-7), 113.71 (qC, C-3), 41.41 (CH_2_, C-11), 26.01 (CH_2_, C-10), 22.59 (CH_3_, C-14); The molecular formula of **2** (HRESIMS *m*/*z* 303.0105, 305.0029 [M + Na]^+^, calc. for C_12_H_13_BrN_2_O).

### 3.4. General Procedure for the Synthesis and Isolation of Compounds **3**–**7**

Both EDC·HCl (0.5 mmol) and HOBt (0.5 mmol) were initially added to the aliphatic acid (0.35 mmol) in DCM (5 mL). After the reaction mixture was stirred at room temperature for 30 min, 6-bromotryptamine (0.25 mmol) dissolved in DCM (7 mL) was added. Subsequently, the mixture was stirred at room temperature for an additional 12 h and dried under reduced pressure. Finally, the residue was washed with water and then redissolved in methanol (10 mL), which was purified on a preparative-HPLC with a gradient elution of MeOH/H_2_O (60%–100%, 60 min, flow 8 mL/min, UV detection at 280 nm) to afford compounds **3** (*t_R_* = 22.6 min), **4** (*t_R_* = 28.6 min), **5** (*t_R_* = 35.6 min), **6** (*t_R_* = 42.5 min), and **7** (*t_R_* = 51.6 min).

*6-Bromo-N-butyryltryptamine* (**3**): Yield 79%, red brown solid; ^1^H-NMR (400 MHz, CDCl_3_) δ (ppm) 9.12 (s, 1HN), 7.49 (d, *J* = 1.7 Hz, 1H), 7.40 (d, *J* = 8.5 Hz, 1H), 7.16 (dd, *J* = 8.4, 1.7 Hz, 1H), 6.93 (s, 1H), 5.93 (s, 1NH), 3.54 (q, *J* = 8.8 Hz, 2H), 2.90 (t, *J* = 6.9 Hz, 2H), 2.09 (t, *J* = 7.4 Hz, 2H), 1.62 (m, 2H), 0.90 (t, *J* = 7.4 Hz, 3H); MS (ESI^+^) *m/z* 330.95, 332.94 [M + Na]^+^.

*6-Bromo-N-pentoyltryptamine* (**4**): Yield 81%, red brown solid; ^1^H-NMR (400 MHz, CDCl_3_) δ (ppm) 8.94 (s, 1HN), 7.49 (d, *J* = 1.7 Hz, 1H), 7.41 (d, *J* = 8.4 Hz, 1H), 7.17 (dd, *J* = 8.5, 1.7 Hz, 1H), 6.95 (s, 1H), 5.77 (s, 1NH), 3.54 (q, *J* = 7.0 Hz, 2H), 2.90 (t, *J* = 7.0 Hz, 2H), 2.11 (t, *J* = 7.5 Hz, 2H), 1.55 (m, 2H), 1.28 (m, 2H), 0.87 (t, *J* = 7.3 Hz, 3H); MS (ESI^+^) *m/z* 344.97, 346.96 [M + Na]^+^.

*6-Bromo-N-hexanoyltryptamine* (**5**): Yield 83%, red brown solid; ^1^H-NMR (400 MHz, CDCl_3_) δ (ppm) 8.95 (s, 1HN), 7.49 (s, 1H), 7.41 (d, *J* = 8.4 Hz, 1H), 7.17 (d, *J* = 8.5 Hz, 1H), 6.95 (s, 1H), 5.76 (s, 1NH), 3.54 (q, *J* = 6.7 Hz, 2H), 2.91 (t, *J* = 6.5 Hz, 2H), 2.11 (t, *J* = 8 Hz, 2H), 1.57 (m, 2H), 1.26 (m, 4H), 0.86 (t, *J* = 6.7 Hz, 3H); MS (ESI^+^) *m/z* 359.01, 360.99 [M + Na]^+^.

*6-Bromo-N-heptanoyltryptamine* (**6**): Yield 80%, white solid; ^1^H-NMR (500 MHz, CD_3_OD) δ (ppm) 7.48 (d, *J* = 1.7 Hz, 1H), 7.46 (d, *J* = 8.4 Hz, 1H), 7.10 (d, *J* = 8.5, 1.8 Hz, 1H), 7.07 (s, 1H), 3.45 (t, *J* = 7.2 Hz, 2H), 2.91 (t, *J* = 7.2 Hz, 2H), 2.13 (t, *J* = 7.5 Hz, 2H), 1.54 (m, 2H), 1.27 (m, 6H), 0.89 (t, *J* = 6.7 Hz, 3H); MS (ESI^+^) *m/z* 373.09, 375.08 [M + Na]^+^.

*6-Bromo-N-octanoyltryptamine* (**7**): Yield 85%, white solid; ^1^H-NMR (500 MHz, CD_3_OD) δ (ppm) 7.48 (d, *J* = 1.7 Hz, 1H), 7.46 (d, *J* = 8.4 Hz, 1H), 7.10 (d, *J* = 8.4, 1.7 Hz, 1H), 7.06 (s, 1H), 3.45 (t, *J* = 7.2 Hz, 2H), 2.91 (t, *J* = 7.2 Hz, 2H), 2.12 (t, *J* = 7.5 Hz, 2H), 1.53 (m, 2H), 1.26 (m, 8H), 0.89 (t, *J* = 7.0 Hz, 3H); MS (ESI^+^) *m/z* 387.11, 389.14 [M + Na]^+^.

### 3.5. Antagonist Activity

All the tested compounds were prepared as follows and stored at −20 °C. Compounds **1**–**7** and the positive control were tested at 10 μM in HBSS buffer (with 20 mM HEPES buffer, pH 7.4) with 0.1% DMSO. Ketanserin tartrate was employed as positive control. According to the previous method, the inhibitory activity was performed in CHO-K1 (Chinese hamster ovary) cells where recombinant human 5-HT_2A_ receptor was expressed, measured by the calcium flux assay [16,17]. The experiments were carried out in triplicates.

## 4. Conclusions

In this paper, two 6-bromotryptamine derivatives with the 5-HT_2A_ receptor antagonist activity, including a new 6-bromo-*N*-propionyltryptamine (**1**) and a known 6-bromo-*N*-acetyltryptamine (**2**) were isolated and characterized from the culture broth of a marine-derived *P. rubra* QD1-2. Moreover, we synthesized a series of new 6-bromotryptamine congeners (**3**–**7**) with different chain lengths (C4–C8) and evaluated their antagonist activity for 5-HT_2A_ receptor. Notably, the preliminary SAR studies indicated that the length of the acyl chain dramatically influenced the antagonist activity of 6-bromotryptamine derivatives. Their antagonist activity increased with elongation of the side chain acyl group (C2–C6), while the activity of compounds **6** and **7** with the side chain longer than that of 6-bromo-*N*-hexanoyltryptamine (C6) significantly declined or disappeared. As the most potent one in this series, 6-bromo-*N*-hexanoyltryptamine (**5**) displayed roughly 4-fold activity enhancement over the parent analogue **1**, which possessed an approximately 70% inhibition activity comparable to the positive control. Overall, 6-bromo-*N*-hexanoyltryptamine (**5**) with a sound antagonist activity for 5-HT_2A_ receptor was identified and our work provides an insight into the further structural optimization of 6-bromotryptamine derivatives for developing potential therapeutic agents to treat neurological disorders.
